# The Efficacy of Muscle Energy and Mulligan Mobilization Techniques for the Upper Extremities and Posture after Breast Cancer Surgery with Axillary Dissection: A Randomized Controlled Trial

**DOI:** 10.3390/jcm13040980

**Published:** 2024-02-08

**Authors:** Omar M. Elabd, Mohammad Etoom, Alhadi M. Jahan, Aliaa M. Elabd, Alaa M. Khedr, Hany M. Elgohary

**Affiliations:** 1 Department of Physical Therapy, Aqaba University of Technology, Aqaba 11191, Jordan; o.elabd@aut.edu.jo; 2 Department of Physical Therapy for Orthopedics and Its Surgeries, Faculty of Physical Therapy, Delta University for Science and Technology, Gamasa 35712, Egypt; 3 Department of Rehabilitation Sciences, Faculty of Applied Medical Sciences, Jordan University of Science and Technology, Irbid 22110, Jordan; 4 School of Rehabilitation Sciences, University of Ottawa, Ottawa, ON K1Y 4W7, Canada; ajaha020@uottawa.ca; 5 Basic Science Department, Faculty of Physical Therapy, Benha University, Banha 13511, Egypt; aliaa.alabed@fpt.bu.edu.eg; 6 Faculty of Physical Therapy, Delta University for Science and Technology, Gamasa 35712, Egypt; alaa.wageeh25@gmail.com; 7 Department of Physical Therapy for Surgery, Faculty of Physical Therapy, Cairo University, Giza 12613, Egypt; hmielgohary@gmail.com; 8 Department of Physical Therapy for Surgery, Faculty of Physical Therapy, Delta University for Science and Technology, Gamasa 35712, Egypt

**Keywords:** mastectomy, manual therapy, upper extremity, rehabilitation

## Abstract

**Background**: Breast cancer surgeries affect the upper extremities and posture. This study aimed to examine the efficacy of muscle energy and Mulligan mobilization techniques on the upper extremities and posture after breast cancer surgery with axillary dissection. **Methods**: A total of 90 female participants who had undergone breast cancer surgery with axillary dissection were recruited and randomly assigned to three groups. Group A received a combination of the Mulligan and muscle energy techniques, while Groups B and C received either the Mulligan or muscle energy techniques for six weeks, respectively. The study measured the shoulders’ range of motion, posture, and upper-extremity disabilities. Outcome measurements were taken at three different time points: baseline, post-intervention, and at eight-week follow-up. **Results**: All the interventions significantly improved the study outcomes. The combination of the Mulligan and muscle energy techniques was significantly better than a single intervention. Mulligan mobilization was superior to the muscle energy techniques in terms of improving the shoulders’ range of motion and disability. The interventions showed a significant effect pre-post-treatment and pre-follow-up but not post-follow-up. **Conclusions**: The Mulligan mobilization and muscle energy techniques have been found beneficial in improving the postural changes and shoulder outcomes after breast cancer surgery with axillary dissection. The superior effectiveness of the combined interventions points out the importance of integrating multiple therapeutic approaches for optimal outcomes. Regular examination and long-term follow-up assessment are important for studying the effect of rehabilitation interventions in people after the late stages of breast surgery.

## 1. Introduction

Breast cancer stands out as the most frequently diagnosed cancer in women, with varied incidence rates and treatment protocols across different regions [1]. In Egypt, breast cancer is the leading cause of cancer-related deaths in women [2]. 

Surgical interventions for breast cancer are considered the first-line treatment for the eradication of tumors [3]. Axillary dissection is one of the standard surgical procedures, performed by incising the axilla [3]. Breast cancer surgeries can cause side effects and lead to complications like shoulder pain, restricted mobility, fibrosis, lymphedema, and biomechanical changes in the shoulders [4,5]. These effects can limit the function of the upper limbs and cause long-term pain. These disorders may become evident either shortly after or several years following breast cancer treatment [5].

Several exercise and manual therapy interventions target shoulder disorders after breast cancer surgery [6,7,8,9]. These interventions include general exercise training such as stretching and strengthening, aerobic exercises, and myofascial release [7]. These interventions show varied levels of benefits on a set of shoulder outcomes such as pain, activity limitations, and participation restriction [8]. However, the interventions show less efficacy for some shoulder kinematics such as shoulder abduction [9]. Furthermore, more high-quality research studies that prescribe exercise and address persistent upper-limb dysfunction are needed to come to conclusions on the most effective interventions [6,9]. Precision in therapeutic intervention is crucial in breast cancer management, emphasizing the pivotal role of physical exercise and rehabilitation in addressing survivorship issues [10,11,12].

We hypothesized that manual therapy interventions targeting shoulder biomechanical malalignment and dysfunction such as Mulligan mobilization with movement (MWM) techniques and muscle energy techniques (MET) could be more beneficial in managing upper-limb dysfunction after breast surgery. Mulligan MWM corrects minor positional faults that occur due to injury or strain, which restrict movement and cause pain [13]. MET is a type of soft tissue or joint manipulation used to treat musculoskeletal dysfunction. MET can help relax and release the muscles and promote the body’s natural healing mechanisms. It is used to normalize the range of motion for joints and can be applied to any joints with a restricted ROM [14]. To our knowledge, there is no previous study assessing the impact of these interventions on the upper extremities in breast cancer surgery. Therefore, the aim of the current randomized controlled trial (RCT) was to assess the efficacy of Mulligan mobilization with movement, muscle energy techniques, or a combination of them on the upper extremities and posture after breast cancer surgery with axillary dissection.

## 2. Materials and Methods

This study is an assessor-blinded parallel RCT reported according to the Consolidated Standards of Reporting Trials (CONSORT) statement [15]. The study was performed in accordance with the ethical standard of the Declaration of Helsinki and approved by the research committees of the authors’ institutions (reference number: F.P.T2207005). The study protocol was registered with ClinicalTrials.gov (NCT05911867). All participants were asked to agree and sign a written consent form.

### 2.1. Participants

The eligibility criteria included participants who had undergone a unilateral axillary dissection mastectomy at least six months prior and had a limited ROM in shoulder flexion and abduction represented by less than 100 degrees. The exclusion criteria were receiving any previous rehabilitation programs for the upper extremities, the presence of chronic musculoskeletal conditions affecting the upper body region, the presence of any type of metastases, the presence of lymphedema, upper-extremity trauma, vascular or musculoskeletal disorders, ongoing anticoagulant therapy, bilateral breast cancer surgery, or locoregional recurrence. The data were collected at the outpatient clinic of the Faculty of Physical Therapy, Delta University for Science and Technology by a surgical oncologist recruited from the Oncology Center of Mansoura University Hospitals, Mansoura, Egypt, between May 2022 and January 2023.

### 2.2. Treatment Procedures

The participants were randomized into one of three groups: Group A received a combination of the Mulligan MWM and MET, Group B received the Mulligan MWM techniques, and Group C received the MET. Experienced physiotherapists delivered the interventions for six weeks, with three sessions per week for all groups. 

#### 2.2.1. Mulligan MWM Techniques

The Mulligan MWM techniques were applied to the shoulders and cervical joints. Regarding the shoulder joints, the therapist glided the humeral head using a belt while the patient moved their shoulder actively through the range. The therapist applies pressure to the scapula in a counter direction. This technique is performed for five sets of five repetitions with one minute between sets in a sitting position [16]. For the cervical structures, the therapist performed self-natural apophyseal glides with force applied to the spinous process of each vertebra using a thumb-over-thumb technique. The patient actively flexed and extended her neck and returned to the neutral position. Passive gliding is maintained in the anterosuperior direction along the facet joint line while flexing or extending the neck through the entire range. Three sets of 10 repetitions were performed following a test trial.

#### 2.2.2. MET

The reciprocal inhibition and post-isometric MET were applied. The therapist stabilized the lateral border of the scapula and passively abducted the arm until the first barrier to motion by applying pressure to the distal humerus. This passive stretch was held for three seconds. The examiner then instructed the participant to attempt to horizontally adduct the test arm at 25% of her maximal effort while the examiner applied manual resistance to the distal humerus to create an isometric contraction lasting for five seconds. The participant then actively abducted her arm for a three-second active-assisted stretch. Four of these application cycles were completed, totaling approximately 60 s, and then the participant rested in a supine position for one minute. The training protocol involves 10 min of training, three times in one session, for a total of 30 min per day following the standard muscle energy techniques for the posterior and inferior shoulder [17].

### 2.3. Outcome Measures

The outcome measurements were measured at three different time points: baseline pre-intervention, post-intervention, and eight weeks after the intervention as a follow-up assessment. 

A—ROM: A digital inclinometer was used to measure the ROM for shoulder flexion and abduction. This tool is considered valid and reliable for shoulder ROM measurement [18]. The measurements were taken three times by the same examiner, and the mean value was recorded as the final measurement.

B—Posture: It was assessed using postural assessment software (PAS/SAPO) version 0.69 to measure the cervical angle and horizontal alignment of the acromion [19]. Anatomical markers were placed on specific points, including the tragus and both acromia, and the women were photographed while standing in a comfortable position to record the angle. PAS/SAPO has been validated and used in breast cancer rehabilitation studies [20].

C—Upper-Extremity Disability: The Quick Disabilities of the Arm, Shoulder, and Hand (DASH) questionnaire was used to assess upper-extremity disability and symptoms. It is a self-reported questionnaire used to assess upper-extremity dysfunction [21]. The questionnaire includes 11 items to measure the difficulty of performing physical activities related to the upper extremities, the severity of pain, and the effect of the problem on social activities, work, and sleep. Each item has five response options ranging from “no difficulty” to “unable to do”. A higher score indicates greater disability. This questionnaire shows good validity and reliability in breast cancer survivors [22].

### 2.4. Sample Size Determination

The required sample size was at least 60 participants to detect a 20% difference in the shoulder flexion and abduction range of motion at a 95% confidence interval and a 5% margin of error. G*Power 3.1 was used to calculate the power sample size. 

### 2.5. Randomization, Allocation Concealment, and Blinding

To assign participants to the treatment groups, a computer-generated table of random numbers was used to randomly allocate patients into one of three groups. The allocation was concealed by using sealed opaque envelopes, and a professional physical therapist who was not involved in the study procedures generated the randomization to ensure an unbiased assignment. The outcome measurements were collected by a well-experienced investigator who was blind to the group assignments and was considered part of the research team.

### 2.6. Statistical Analysis

Statistical analysis was performed using SPSS software version 21.0 (Chicago, IL, USA). Descriptive statistics were calculated for the three groups at three time intervals: baseline, post-intervention, and an eight-week follow-up assessment. The Shapiro–Wilk test was used to check the normal data distribution. One-way ANOVA was used to assess the difference in the demographic characteristics and all outcome measures at baseline between the three groups. Two-way repeated ANOVA and post hoc Tukey tests were used with one between-subject factor [Group A vs. Group B vs. Group C] and one within-subject factor [pre vs. post vs. follow-up] for all the outcome measures. *p*-values lower than 0.05 were considered statistically significant.

## 3. Results

**Figure 1:**
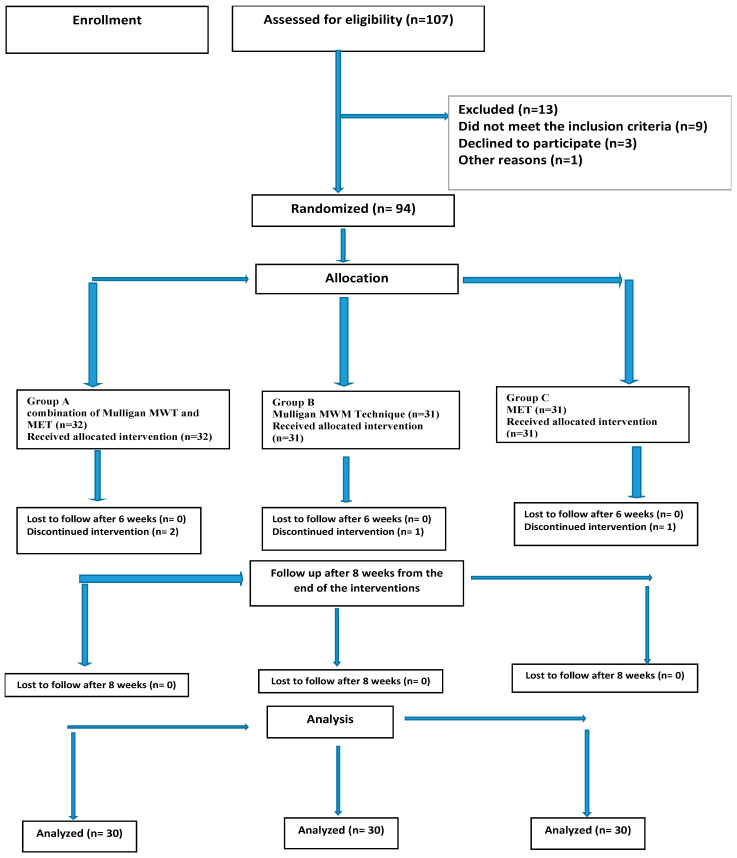
Consort flowchart of participant recruitment, allocation, and follow-up. MET; muscle energy technique, MWM; Mulligan mobilization with movement.

Out of 107 patients screened for the eligibility criteria, only 93 were eligible and randomly assigned into one of three groups. Four patients were unable to complete the study due to transportation issues. The patient flow diagram throughout the study is shown in Figure 1. Table 1 shows the baseline characteristics of the three study groups, and there was no significant difference between the groups at the baseline.

All the interventions significantly improved the study outcomes. The post hoc analysis shows that Group A was significantly better than Group B and Group C (Table 2). Group B was significantly better than Group C in improving the shoulder ROM and Quick DASH (Table 2). A significant interaction between the time and group was found for all the outcome measures. The outcomes in all three groups were significantly improved t pre-post-intervention and pre-follow-up. However, the mean differences between post-intervention and the follow-up were not statistically significant (Table 3).

## 4. Discussion

The current study aimed to examine the efficacy of manual therapy interventions for posture and upper-extremity kinematics and disability in women with breast cancer. The manual therapy interventions showed a significant effect on the study outcomes. The study included participants who had undergone a unilateral axillary dissection mastectomy at least 6 months prior. We recruited participants 6 months after because most of the rehabilitation research has included participants at an acute stage after breast surgery [23]. Furthermore, there is a high prevalence of long-term complications such as upper-extremity pain and disability after these surgeries [24]. However, it is important to study the effect of rehabilitation interventions in people after the late stages of breast surgery. In fact, the clinical guidelines recommend starting upper-extremity exercise after surgery as early as possible [23,25]. The current evidence has found obvious advantages of early physical rehabilitation in comparison with rehabilitation at the late stages after breast cancer surgery [26,27]. The current study findings recommend manual therapy interventions, specifically MWM and MET, for the upper extremities and posture in women after the last stage of breast cancer surgeries.

The study outcomes at the baseline show slight limitations in the shoulder ROM but nevertheless high levels of upper-extremity disability. Although the shoulder ROM measurements were in the near-functional range [28], this was not reflected in the upper-extremity disability, maybe due to the assessment areas in Quick DASH of pain, abnormal sensation, and sleep, which measure upper-extremity disability beyond ROMs [21]. This supports the recommendations on the importance of continuing shoulder exercises until the full range of motion is reached [23,25].

The study found significant effects of the Mulligan mobilization and muscle energy techniques, with higher benefits with a combination of the two. Furthermore, the benefits of the intervention were retained at the 8-week follow-up. The improvements in the MWM group could be attributed to its biomechanical effects. The MWM corrects the joint arthrokinematics by repositioning the joints, causing them to track normally [29,30,31]. The mechanical benefits may include increasing the fiber glide when specific movements stress specific parts of the capsular tissue, improving the normal extensibility of the shoulder capsule, stretching tightened soft tissue, and normalizing the scapulohumeral rhythm, as investigated in different shoulder conditions [29,30,31]. On the other hand, the improvements in the MET could be attributed to its relaxation effect according to neurophysiological mechanisms based on the stimulation of peripheral mechanoreceptors and the inhibition of nociceptors. Activation of the apical spinal neurons as a result of the effect of joint mobilization on the peripheral mechanoreceptors produces presynaptic inhibition of the nociceptive afferent activity, reducing pain, which could result in improving the shoulder biomechanics, ROM, and functional ability [32].

A combination of MWM and MET resulted in significantly better results. A possible explanation is the upper-extremity and posture abnormalities were caused by both muscle and joint impairments. Biomechanical analyses [33,34] found significant alterations in the scapulohumeral rhythm, alterations in the upper-extremity muscle activity and control, and a suboptimal scapular tilt. These wide impairments required integrated rehabilitation programs at the joint and muscle levels. The muscles were better influenced by MWM than MET in term of improving the shoulder ROM and function. This is in line with a previous study comparing Mulligan mobilization and MET in shoulder conditions [35]. We strongly recommend rehabilitation specialists target both muscles and joints during the rehabilitation process after breast cancer surgery.

Axillary dissection mastectomy is correlated with high shoulder disability [36,37]. One of the reasons for this is postural malalignment as a complication of the surgery. Women who undergo axillary dissection mastectomy adopt a compensatory posture represented by contractures in the cervical and scapular girdle, head tilting, and elevation of the shoulders to avoid pain and hide the absence of the breasts [36,37]. The current study data confirmed postural malalignment after axillary dissection mastectomy. The baseline data show cervical angle and acromia position malalignment. Our study interventions show a significant effect on the cervical and acromia posture. Few studies have assessed the effectiveness of rehabilitation interventions on posture after breast cancer surgeries, and therefore there is a need to address the effect of different interventions on posture [20].

This study has some limitations. This was a single-center RCT, which may limit the generalizability of the results. Moreover, quality of life was not assessed. In fact, the quality of life outcomes were influenced by upper-extremity status in previous reports [12,38]. However, assessing the quality of life is important to confirm the positive effects of manual therapy interventions.

## 5. Conclusions

The current study provides a robust RCT finding that MWM and MET are beneficial for shoulder ROM, disability, and posture in the late stage after breast cancer surgery with axillary dissection. The superior effectiveness of the combined interventions points out the importance of integrating multiple therapeutic approaches for optimal outcomes. Regular examination and long-term follow-up assessment are important to study the effect of rehabilitation interventions in people after the late stages of breast surgery.

## Figures and Tables

**Table 1:** Patients’ demographic characteristics and baseline outcome measures.

Variable/Outcome	Group (A) Mean ± SD	Group (B) Mean ± SD	Group (C) Mean ± SD	*p*-Value
Age (year)	57.2 ± 4.92	57.77 ± 4.16	57.47 ± 3.98	0.88
Weight (kg)	74.03 ± 9.96	75.1 ± 9.95	76.2 ± 7.25	0.66
Height (m)	1.69 ± 0.06	1.66 ± 0.08	1.68 ± 0.06	0.39
BMI (Kg/m^2^)	26.11 ± 3.43	27.31 ± 3.67	27.23 ± 2.90	0.31
Shoulder ROM:				
Shoulder flexion (degree)	105.97 ± 15.68	103.6 ± 14.57	105.5 ± 13.73	0.80
Shoulder abduction (degree)	102.37 ± 14.35	103.37 ± 16.89	102.37 ± 14.35	0.96
Posture alignment:				
Cervical angle (degree)	38.67 ± 3.67	39.7 ± 3.30	39.2 ± 3.96	0.55
Horizontal alignment (mm)	3.74 ± 0.64	3.57 ± 0.72	3.86 ± 0.61	0.24
Upper-extremity disability:				
Quick DASH	79.43 ± 6.82	76.83 ± 8.53	76.37 ± 9.72	0.32

**Table 2:** Comparing mean values of the study outcomes at pre-/post-treatment and follow–up for Groups A, B, and C. DASH; Disabilities of the Arm, Shoulder and Hand, SD; standard deviation. * Significant at *p* < 0.05.

Outcome	Study Groups	Pre-	Post-	Follow-Up	*p*-Value	Between-Group Variation (F, P)	Post Hoc
		Mean ± SD	Mean ± SD	Mean ± SD			
A—Shoulder ROM:							
Shoulder flexion (degree)	Group A	105.97 ± 15.68	173.8 ± 3.99	176.6 ± 2.66	<0.001 *	(F [24,7] = 535.77, *p* ≤ 0.001 *)	A > B A > C B > C
	Group B	103.6 ± 14.57	157.6 ± 7.11	156.43 ± 5.99	<0.001 *		
	Group C	105.5 ± 13.73	122.77 ± 11.91	119.6 ± 13.18	<0.001 *		
Shoulder abduction (degree)	Group A	102.37 ± 14.35	174.5 ± 3.29	177.13 ± 2.52	<0.001 *	(F [24,7] = 592.89, *p* ≤ 0.001 *)	A > B A > C B > C
	Group B	103.37 ± 16.89	158.77 ± 4.91	159.93 ± 4.54	<0.001 *		
	Group C	102.37 ± 14.35	117.17 ± 12.44	117.87 ± 12.04	<0.001 *		
B—Posture alignment:							
Cervical angle (degree)	Group A	38.67 ± 3.67	59.1 ± 3.46	59.6 ± 3.18	<0.001 *	(F [24,7] = 225.29, *p* ≤ 0.001 *)	A > B A > C
	Group B	39.7 ± 3.30	49.69 ± 4.57	49.43 ± 4.31	<0.001 *		
	Group C	39.2 ± 3.96	44.93 ± 5.95	45.17 ± 6.26	<0.001 *		
Horizontal alignment of acromion (mm)	Group A	3.74 ± 0.64	2.28 ± 0.65	2.18 ± 0.60	<0.001 *	(F [24,7] = 277.87, *p* ≤ 0.001 *)	A > B A > C
	Group B	3.57 ± 0.72	2.53 ± 0.24	2.49 ± 0.21	<0.001 *		
	Group C	3.86 ± 0.61	2.79 ± 0.81	2.70 ± 0.80	<0.001 *		
C—Upper-extremity disability:							
QuickDASH	Group A	79.43 ± 6.82	17.43 ± 4.44	16.7 ± 4.74	<0.001 *	(F [24,7] = 943.33, *p* ≤ 0.001 *)	A < B A < C B < C
	Group B	76.83 ± 8.53	41.13 ± 7.35	40.67 ± 6.61	<0.001 *		
	Group C	76.37 ± 9.72	51.1 ± 8.38	51.17 ± 7.86	<0.001 *		

**Table 3:** Mean differences and confidence intervals at study time point (pre, post, follow-up). CI; Confidence interval. DASH: Disabilities of the Arm, Shoulder and Hand. * Significant at *p* < 0.05.

Outcome	Study Groups	Time Measurement	Mean Change (95% CI for Differences)	*p*-Value	Time × Groups (F, p)
A—Shoulder ROM:					
Shoulder flexion (degree)	Group A	Pre * Post	67 (62–73.66)	<0.001 *	(F [24,7] = 64.152, *p* ≤ 0.001 *)
		Pre * Follow-up	70.63 (64.81–76.46)	<0.001 *	
		Post * Follow-up	2.8 (−3.02–8.63)	0.49	
	Group B	Pre * Post	54 (47.86–60.14)	<0.001 *	
		Pre * Follow-up	52.83 (46.69–58.98)	<0.001 *	
		Post * Follow-up	−1.16 (−7.31–4.98)	0.89	
	Group C	Pre * Post	17.27 (9.29–25.24)	<0.001 *	
		Pre * Follow-up	14.1 (6.12–22.08)	<0.001 *	
		Post * Follow-up	−3.17 (−11.15–4.81)	0.61	
Shoulder abduction (degree)	Group A	Pre * Post	72.13 (66.82–77.44)	<0.001 *	(F [24,7] = 76.18, *p* ≤ 0.001 *)
		Pre * Follow-up	74.77 (69.46–89.08)	<0.001 *	
		Post * Follow-up	2.63 (−2.68–7.94)	0.47	
	Group B	Pre * Post	55.4 (48.94–61.86)	<0.001 *	
		Pre * Follow-up	56.57 (50.11–63.02)	<0.001 *	
		Post * Follow-up	1.17 (−5.29–7.62)	0.90	
	Group C	Pre * Post	14.8 (6.81–22.79)	<0.001 *	
		Pre * Follow-up	15.5 (7.51–23.49)	<0.001 *	
		Post * Follow-up	0.7 (−7.29–8.69)	0.97	
B—Posture Alignment:					
Cervical angle	Group A	Pre * Post	20.43 (18.32–22.55)	<0.001 *	(F [24,7] = 30.16, *p* ≤ 0.001 *)
		Pre * Follow-up	20.93 (18.82–23.05)	<0.001 *	
		Post * Follow-up	0.5 (−1.62–2.62)	0.83	
	Group B	Pre * Post	9.93 (7.41–12.46)	<0.001 *	
		Pre * Follow-up	9.73 (7.21–12.26)	<0.001 *	
		Post * Follow-up	−0.2 (−2.72–2.32)	0.98	
	Group C	Pre * Post	5.73 (2.36–9.11)	<0.001 *	
		Pre * Follow-up	5.97 (2.59–9.34)	<0.001 *	
		Post * Follow-up	0.23 (−3.14–3.61)	0.99	
Horizontal alignment of acromia (mm)	Group A	Pre * Post	−3.08 (−3.36–2.79)	<0.001 *	(F [24,7] = 27.45, *p* ≤ 0.001 *)
		Pre * Follow-up	−3.04 (−3.22–2.76)	<0.001 *	
		Post * Follow-up	0.04 (−0.24–0.32)	0.94	
	Group B	Pre * Post	−1.46 (−1.85–1.07)	<0.001 *	
		Pre * Follow-up	−1.56 (−1.94–1.17)	<0.001 *	
		Post * Follow-up	−0.09 (−0.48–0.29)	0.82	
	Group C	Pre * Post	−1.07 (−1.53–0.61)	<0.001 *	
		Pre * Follow-up	−1.15 (−1.61–0.69)	<0.001 *	
		Post * Follow-up	−0.08 (−0.55–0.38)	0.89	
C—Upper-extremity disability:					
Quick DASH	Group A	Pre * Post	−62 (−65.35–58.65)	<0.001 *	(F [24,7] = 67.77, *p* ≤ 0.001 *)
		Pre * Follow-up	−62.73 (−66.08–59.39)	<0.001 *	
		Post * Follow-up	−0.73 (−4.08–2.61)	0.86	
	Group B	Pre * Post	−35.7 (−40.34–31.06)	<0.001 *	
		Pre * Follow-up	−36.17 (−40.81–31.53)	<0.001 *	
		Post * Follow-up	−0.47 (−5.11–4.17)	0.97	
	Group C	Pre * Post	−25.27 (−30.61–19.92)	<0.001 *	
		Pre * Follow-up	−25.2 (−30.55–19.85)	<0.001 *	
		Post * Follow-up	0.07 (−5.28–5.41)	0.99	

## Data Availability

The data that support the findings of this study are available from the first author [O.A.] upon reasonable request.
